# SOCIETAL, OCCUPATIONAL, AND ECONOMIC CONSIDERATIONS FOR WOMEN WITH (M)INOCA: A NARRATIVE REVIEW

**DOI:** 10.13075/ijomeh.1896.02527

**Published:** 2025

**Authors:** Sasha Voznyuk, Emilie T. Théberge, Mahraz Parvand, Tara L. Sedlak

**Affiliations:** 1 University of British Columbia, Department of Medicine, Vancouver, British Columbia, Canada; 2 Vancouver Coastal Health, Vancouver, British Columbia, Canada; 3 BC Women's Hospital, Vancouver, British Columbia, Canada

**Keywords:** women, employment, microvascular angina, MINOCA, return-to-work, coronary vasospasm

## Abstract

Cardiovascular disease is one of the leading causes of mortality in women, despite underrepresentation in the medical literature. Women have higher rates of ischemia and no obstructive coronary artery disease (INOCA) and myocardial infarction with no obstructive coronary artery disease (MINOCA) compared to men. The aim of this review is to describe the occupational, economic, and psychosocial factors which disproportionately impact women with (M)INOCA. Relevant databases including MEDLINE, EMBASE, and CINAHL were searched using keywords related to ischemic heart disease, nonobstructive coronary syndromes, (M)INOCA, women's health, questionnaires and surveys, cohort studies, workplace outcomes, and health costs. This narrative review includes key findings from 50 articles that fit the inclusion criteria. Sex-based differences among patients with nonobstructive coronary syndromes are discussed in the context of health care service utilization, working status, and job characteristics. Despite lower mortality rates, nonobstructive coronary syndromes are associated with a large burden of clinical, functional, and economic implications. Women face significant morbidity, productivity losses, and early exit from the workforce. Existing literature focuses on ischemic heart disease as an entity without specific attention to (M)INOCA, and recent health economic studies are lacking. Despite growing recognition of (M)INOCA endotypes and improved diagnostic modalities, its economic and societal impacts remain under-researched, highlighting the need for validated tools to measure work impairment. Collaborative efforts including workplace and employer participation are needed to address work-related outcomes. Researchers and institutions need to consider the interplay of sex-based differences and societal impacts on women.

## Highlights

- Nonobstructive coronary disease, i.e., myocardial infarction with no obstructive coronary artery disease (M)INOCA, disproportionately affects women.
- Women with (M)INOCA suffer from increased emotional and physical stress.
- Greater morbidity and symptom burden impairs participation in the workforce.
- Effective tools for evaluating productivity losses in this population are lacking.

## INTRODUCTION

Cardiovascular disease (CVD), broadly including heart disease and stroke, is one of the leading causes of death among Canadian women [1]. Ischemic heart disease (IHD) is an umbrella term that includes syndromes such as stable angina, unstable angina, acute coronary syndrome (ACS), non-ST-elevation myocardial infarction (NSTEMI), and ST-elevation myocardial infarction (STEMI) [2]. Many Canadian women have at least 1 cardiovascular risk factor [3] and almost 45% of American women have clinical CVD by the time they reach the age of 20 years [4]. The absolute mortality rate among women with heart disease across Canada was 17.6% in 2018, of which coronary artery disease (CAD) was the most common cause of cardiovascular mortality [3], although this data is not subdivided into obstructive and nonobstructive causes.

Obstructive CAD is defined as ≥50% stenosis in any epicardial artery. In contrast, patients with ischemia and no obstructive coronary artery disease (INOCA) have <50% stenosis [2,5,6]. The pathophysiology of INOCA often involves myocardial perfusion defects due to coronary microvascular dysfunction (CMD) [2,6]. Endotypes of INOCA include microvascular angina, vasospastic angina, and mixed vasomotor angina. The etiology of myocardial infarction with no obstructive coronary artery disease (MINOCA), i.e., myocardial infarction (MI) in the absence of at least 50% stenosis in any epicardial artery, includes CMD, epicardial vasospasm, coronary plaque disruption, spontaneous coronary artery dissection (SCAD), and other non-ischemic causes such as Takotsubo cardiomyopathy and myocarditis [7].

Women's Heart Centers (WHCs) or Women's Heart Programs (WHPs) address sex-specific cardiac risk factors, symptoms, etiologies, and presentations. They provide centralized access to multidisciplinary care teams and specialized diagnostic procedures to identify women with (M)INOCA [8,9]. They offer a holistic approach involving physicians and nurse practitioners with additional training in women's heart health, mental health professionals, social workers, dietitians, and peer support leaders [8,9]. Currently, the Canadian provinces of British Columbia (BC), Ontario, Quebec, and Nova Scotia have dedicated WHCs [9].

Access to dedicated WHCs has improved accessibility to diagnostic testing in urban areas, allowing for the recognition of (M)INOCA endotypes. For example, at a WHC in BC, Canada, 71.4% of women with no obstructive CAD on angiography received a probable or definite diagnosis of coronary microvascular disease or vasospasm through diagnostic testing with cardiac magnetic resonance imaging or invasive coronary reactivity testing [8] and increased to 78% following 3-year attendance [10]. Multiple mechanisms may coexist in (M)INOCA, and this heterogeneity makes high-quality clinical trials and definitive guidelines challenging. For the purposes of this paper, these etiologies will be grouped into a singular category because the societal and economic discourse on (M)INOCA as an entity is limited.

**Figure 1.**
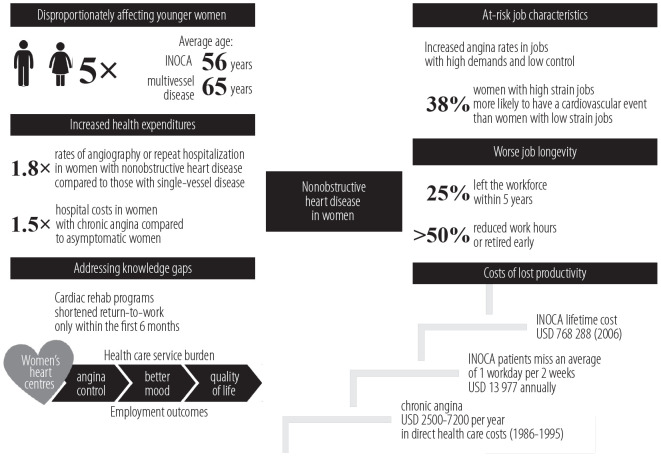
The intersection between societal, occupational, and economic factors among women with nonobstructive disease INOCA – ischemia and no obstructive coronary artery disease.

The aim of this narrative review is to outline the societal and occupational impacts of nonobstructive heart disease in women, emphasizing how sex-based differences in heart disease disproportionately affect women's psychosocial wellbeing and interfere with their participation in the workforce. This paper identifies factors which both directly and indirectly mediate the effects of (M)INOCA on lost productivity and increased health service utilization, while highlighting the relative absence of similar data in the Canadian context (Figure 1). Narrative methodology was chosen because the intent of this paper is to provide a cohesive, high-level overview.

## METHODS

**Figure 2.**
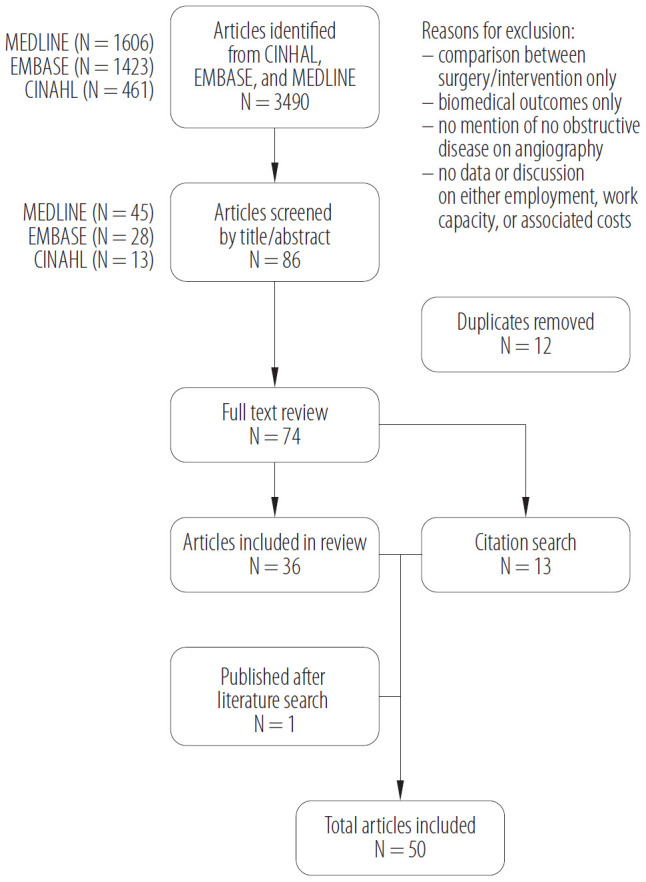
Search strategy flow chart of the review from database inception to May 20, 2023, conducted in British Columbia, Canada, on articles published in the English language

Relevant databases including MEDLINE, EMBASE, and CINAHL were searched using keywords related to ischemic heart disease, nonobstructive coronary syndromes, (M)INOCA, women's health, questionnaires and surveys, cohort studies, workplace outcomes, and health costs. The search was performed from inception until May 20, 2023. The search terms included the following as MeSH headings or keywords: “INOCA” OR “MINOCA” OR “angina pectoris” OR “microvascular angina” OR “coronary vasospasm”; AND “work performance” OR “work capacity” OR “work resumption” OR “employment” OR “disability” OR “return-to-work” OR “job performance” OR “vocational”; AND “women.” The earliest publication was from October 1969 and the most recent was in April 2023. Inclusion criteria included original research with a primary population of women with diagnosed or presumed INOCA, MINOCA, or unspecified ischemic heart disease; articles that explored sex-based differences of the aforementioned; evaluation of work-related outcomes, including performance, return to work, or workplace characteristics among women with IHD; validation of a questionnaire in women with IHD that attempted to capture some aspect of work capacity, including studies in which it was a secondary outcome; and syntheses or review articles on (M)INOCA that were published in the English language. The reference lists of relevant articles were searched. One of the identified articles had a sequential, longitudinal follow-up study published following completion of the literature search. Thus, it was also included in the review. In total, 50 articles that fit the inclusion criteria are discussed in this narrative review (Figure 2).

The grey literature was searched to identify existing occupational or work productivity questionnaires. Select primary publications and secondary analyses related to the economic or occupational impact of IHD were included as well, as there were few publications on (M)INOCA specifically. Only 6 articles directly described the economic and/or occupational impact of (M)INOCA [11–16]. Several articles mentioned the clinical and functional implications of (M)INOCA [8,11,12,14,16–19] or drew comparisons to obstructive CAD [12,20]. Studies with data on sex differences were included as well, although women were largely underrepresented. There are no studies on non-binary individuals or trans women with (M)INOCA. To the authors' knowledge, there is no literature characterizing the degree of workplace impairment in women with (M)INOCA, nor its broad economic or societal impacts.

## RESULTS

### Sex-based differences in heart disease

Clinical and epidemiological research on heart disease has predominantly focused on men, who have a higher prevalence and incidence of ischemic disease [1,2]. However, the overall mortality and re-hospitalization rates of women due to cardiovascular diseases are higher than men, especially among younger women [21,22]. Women are also disproportionately affected by nonobstructive syndromes compared to men, who instead have higher rates of obstructive disease [2,7,23,24]. In a cohort of MI patients aged 18–55 years, women were 5 times more likely to have (M)INOCA compared to men [25]. In a multi-center North American observational study of patients with SCAD, women made up 89% of the population [26]. Women have higher rates of microvascular angina than men, which may be missed unless specific testing modalities are used. Among patients with stable chest pain who had no evidence of coronary obstruction, a systematic review with aggregate data from over 440 000 patients across 20 studies estimated the prevalence of microvascular angina based on positive non-invasive testing to be 43%. Within this subgroup, women outnumbered men by a factor of 2.5 [24]. In a retrospective cohort study in Denmark with >11 000 patients [27], women made up two-thirds of the patient population with angina and nonobstructive CAD. These findings align with data from 13 695 adults in BC with stable angina following coronary angiography, where the rates of nonobstructive CAD in women (11.5%) were more than double the rates seen in men (5.5%) [23]. The risk of cardiovascular events in women with stable angina and nonobstructive CAD was almost 3 times higher than men with this diagnosis in the first year after coronary angiography [23]. Stable angina was associated with a 52% and 89% increase in cardiovascular events in patients with normal coronary arteries and diffuse nonobstructive CAD, respectively, compared to patients without ischemic heart disease [27].

The economic impact of (M)INOCA may be partially explained by the distribution of the affected demographic. On average, women with nonobstructive disease are younger than women with obstructive CAD. Based on data from the Women's Ischemia Syndrome Evaluation (WISE), the average age of women with INOCA was 56 years, while women with single-, double-, and multi-vessel disease were 61–65 years on average [12]. A meta-analysis of 28 studies on MINOCA found a median age of 55 years [28], which is younger than national averages for age of retirement. In addition, patients with chronic angina were 3 times more likely to be on disability and 50% more likely to leave the workforce before their retirement age [15]. The debilitating symptoms of (M)INOCA often precede formal diagnosis, and their onset may be seen while women are still active participants in the workforce. Presumably, low-yield diagnostic tests and ineffective treatments may lead to unnecessarily high health system costs, while symptom burden may lead to presenteeism, absenteeism, and early retirement or medical leave.

### Health service utilization

Women with (M)INOCA may pose a significant strain on healthcare service utilization. Although their rates of major cardiac events were lower compared to women with obstructive CAD, drug therapy for women with nonobstructive CAD totaled 32.6% of their 5-year cumulative cardiovascular costs, which is higher than in women with multi-vessel disease [12]. The rates of repeat angiography or hospitalization for angina were 1.8-fold higher for women with nonobstructive CAD compared to women with single-vessel disease within 1 year of follow-up [12]. Women with persistent chest pain were more likely to undergo repeat angiography [29] and had hospitalization costs that were 1.5 times higher compared to asymptomatic women [12]. Women with normal coronary arteries were 4 times more likely to be readmitted for chest pain within 6 months and thrice as likely to experience a cardiac event within the first year after angiography compared to men [23,30].

The estimated lifetime cost of cardiovascular services for a woman with nonobstructive disease in 2006 was USD 768 288, extrapolated from acute care and outpatient services, diagnostics, reduced work and productivity hours, and medication costs in the USA [12]. In comparison, the average lifetime estimates for women with single-vessel and triple-vessel disease were USD 1 001 493 and USD 1 051 302, respectively [12]. Although obstructive disease carries a higher cumulative cost burden, when considering the younger age and greater life expectancy of women with (M)INOCA, this economic impact is substantial. While the authors acknowledge the limitations of extrapolating these outdated estimates in the current health care context, this study highlights an important comparison of sex-specific costs between nonobstructive versus obstructive disease.

The authors did not identify a similar estimate for Canadian women, nor any current data pertaining to (M)INOCA in the literature. However, diagnostic cardiac catheterization bills the BC Medical Services Plan (MSP) USD 245 per service, with USD 2 714 687 in cumulative expenditures for 2020/2021. In the same fiscal period, USD 33 154 was billed to MSP for emergency visits requiring cardiologist assessment [31,32]. This is important when considering that women with (M)INOCA have higher rates of utilizing these services, yet receive minimal diagnostic or therapeutic benefit from them. Nationally, estimated hospital admission costs associated with stable angina for 1 patient between the ages of 18–59 years is approx. USD 1883. For each patient with stable angina undergoing coronary angiogram, the cost increases to USD 3522. These estimates are available through the national health care data provided by the Canadian Institute for Health Information, although it is not subdivided based on sex or chest pain etiology. The proportion of interventional services utilized by (M)INOCA patients or their degree of health service access is not well-documented, which precludes crude estimations on cost burden.

### Functional impairment

There is evidence from mixed-sex studies that (M)INOCA patients have significant impairments in occupational, social, and functional domains [13,14]. One study found significantly lower exercise capacity and quality of life compared to healthy controls, as measured by the *Short Form Survey* (SF-36) [18]. Interestingly, they also scored worse on domains related to mental health and vitality compared to patients with CAD [18]. More than half of INOCA patients had to reduce 5 or more leisurely, social, or instrumental activities due to chest pain [14]. Ischemia and no obstructive coronary artery disease symptoms were negatively associated with the ability to do work, with 70% of patients reporting adverse effects on their work life. More than half reduced work hours or retired early, and up to 80% left the workforce altogether due to chest pain [11,14].

Functional disability, defined as a metabolic equivalents (METS) score of ≤4.7 on the *Duke Activity Status Index*, has been reported by nearly half of women with INOCA, and living with INOCA was positively associated with a significant reduction in functional capacity of approx. 3 METS, or the equivalent of losing the ability to do light housework or walk 1 block [11,12]. Among patients with stable angina undergoing scheduled angiography, health-related quality of life was significantly worse in women than men [19]. There was no difference between those who were ultimately diagnosed with obstructive compared to nonobstructive CAD, suggesting this association is driven by symptom severity rather than the extent of cardiac disease [19].

Individuals may need to demonstrate evidence of severe mental or physical impairments interfering with their ability to perform activities of daily living that is expected to continue beyond 2 years in order to qualify for disability benefits across certain provinces in Canada. Insurance providers may request a functional ability evaluation, including a musculoskeletal examination, tests of fine motor skills, higher-level cognitive abilities, and functional tasks. In addition to a lack of standardized diagnostic criteria or an agreeance on shared terminology for (M)INOCA [33], there are no standardized tools for evaluating the degree of workplace impairment in (M)INOCA patients. Consequently, symptomatic patients with suspected or formally diagnosed (M)INOCA risk facing delays in accessing benefits. Furthermore, their degree of disability may be underappreciated in the absence of reproducible physiological changes, or if they are evaluated by a provider who is unfamiliar with (M)INOCA syndromes.

### Impact of job strain and job demands

While there is interplay between employment, personal profiles, and the frequency and severity of angina symptoms in women, the data is conflicting, and most is focused on ischemic disease as a collective entity rather than on (M)INOCA. There are some associations between financial or work stressors and symptom severity among women with nonobstructive disease, although socioeconomic status may be an intermediary factor. It is challenging to extract data from cohorts that do not stratify their participants on the basis of CAD etiology, which may also be explained by limited access to more sophisticated diagnostic procedures and the classification of heart disease into a singular category for research purposes. The association between job characteristics and angina symptoms or cardiovascular events is still of relevance to (M)INOCA and can be used to identify vulnerable sectors and individuals at risk.

Moderate-to-severe financial stress was reported in >60% of women with clinical symptoms of ischemia but no coronary disease on angiography who were enrolled in an independent cohort study titled WISE-Coronary Vascular Dysfunction (WISE-CVD), which did not feature any of the same participants in the previously discussed WISE study [16]. Financial stress was consistently associated with cardiac symptoms. However, stress at home or work was less reliably predictive of cardiac symptoms among this cohort, in contrast to the original WISE study, where participants tended to represent a lower socioeconomic class [16,20].

In a mixed-sex prospective cohort study, high job strain was associated with a 2.2-fold cardiovascular mortality risk compared to low job strain [34]. Data from the Women's Health Study with >22 000 female health professionals found that women with high strain jobs or active jobs were 38% more likely to experience their first cardiovascular event relative to women with low strain jobs [35]. High job strain is characterized by high work demands and low job control, while active jobs have high demands and high control [34,35]. Questionnaire data from >7000 employees aged 40–60 years found that high job demands, low job control, high physical strain at work, and work fatigue were positively associated with angina symptoms in women, as measured on the *Rose Angina Questionnaire* [36,37]. These associations were independent of socioeconomic variables [35–37], and only 11–26% of the relationship between job strain and disease could be attributed to depression and anxiety [35]. It is noteworthy that women with (M)INOCA and pre-existing depression tend to score higher on angina symptom severity [10]. Other risk factors contributed even less to this association, suggesting that traditional lifestyle factors and comorbidities are insufficient to explain the relationship between work stress and CVD [35].

High effort-reward imbalances at work were associated with an increased risk for recurrent coronary events over a 4-year period after returning to work following MI. The effects were even stronger for women, although this finding must be interpreted with caution as the particular prospective study was underpowered [38]. A meta-analysis of 11 European cohort studies also found an increased risk of CAD with effort-reward imbalance, independent of job strain [39]. Job strain independently predicted delayed return to work after ACS as measured using the 24-item *Job Content Questionnaire*, which has been implemented internationally in relation to job strain and CVD and validated in select Japanese samples [40]. Women were underrepresented here as well, constituting slightly >5% of the cohort [40]. Conversely, job strain and job dissatisfaction were not found to be associated with an increased risk of IHD among a cohort of over 18 000 public service workers in Denmark, 79% of whom were women [41]. Women employed outside the home were 30% less likely to have IHD compared to women who stayed at home [42]. However, baseline employment data in this study was collected between the ages of 45–64 years, which may not account for occupation in earlier years. Therefore, self-selection into either of these groups could be explained by health or functional status as a confounder [42].

It is premature to say that women with (M)INOCA have a proclivity to work in a specific environment that in turn imparts a heightened risk. It would be valuable to collect employment history information from women being investigated for (M)INOCA and explore whether they are more likely to report increased job strain and pressure, either past or present. Because (M)INOCA is less strongly associated with traditional risk factors compared to obstructive disease [27,43–45], describing this relationship further may add to the breadth of evidence around the directionality of job characteristics and heart disease, by removing some of the confounding effects of genetic risk and lifestyle factors.

### Productivity losses and return to work

Most publications on workplace productivity are focused on patients broadly after MI or ACS, which must be interpreted with caution in the context of (M)INOCA but may suggest outcome measures of interest for future studies. Compared to matched cohorts, the annual earning losses were 8.1% in the 3 years following an acute MI, and the number of patients employed during this period decreased by 5% [46]. On average, patients with ACS lost 59 working days in the year following the initial event as measured by the *Productivity Cost Questionnaire*, which relies on a 4-week recall period [47]. Secondary analysis of data from a 2004–2006 international clinical trial evaluating the medication ranolazine in non-ST-elevation ACS found that patients with daily angina incurred USD 4000 in resource utilization and incremental costs over a period of 8 months, more than double that of patients with no angina, with the majority of costs attributable to hospitalizations and diagnostic testing [48].

In acute MI, there is a distinct event that can precipitate a decline in function. Based on data from over 20 000 individuals from nationwide Danish registries, although most returned to work after an MI, approx. one-quarter became detached from employment within the following year and relied on social benefits for support [49]. This was particularly pronounced in younger patients (30–39 years) and those of lower socioeconomic status [49]. A longitudinal study following over 9300 patients for 1-year post-MI found that 10% experienced an adverse change in employment, which was associated with higher depression and lower quality of life scores [50]. Conversely, it is less defined at which point a patient with INOCA becomes unwell. Unfortunately, many women enter WHCs during their disease course, limiting the ability to assess employment changes throughout their illness period. The impact of cardiac programs may demonstrate different results in (M)INOCA, because it becomes an entity of chronic disease management rather than an episodic rehabilitative program.

An Australian microsimulation model used national survey data to analyze the economic impacts of lost productive life years due to IHD in individuals aged 45–64 years. Data was de-identified and was not stratified based on sex or obstructive versus nonobstructive etiologies [51]. Productive life years were defined as the length of time an employee is in the workforce prior to reaching the age of retirement. Variables in this model included labor force participation, personal income, government revenue, and welfare payments. A projected 6700 people were out of the labor force in 2015 due to IHD, with an expected increase to 8100 in 2030. The projected national cost in 2015 due to lost labor was USD 284 million and is expected to increase to USD 426 million by 2030 [51].

The mean score on the *Work Limitations Questionnaire* (WLQ), a measure of chronic health conditions on worker productivity, within a cohort of 66 INOCA patients showed significant work limitations. They missed an average of 1 workday every 2 weeks, equating to an estimated annual cost of USD 13 977 per patient, subdivided as USD 9819 due to absenteeism and USD 4158 due to presenteeism [13]. After 5 years of follow-up, 18–25% of patients with angina and normal coronary arteries or diffuse nonobstructive CAD left the workforce prematurely [15]. There is evidence to support cardiac rehabilitation programs in facilitating return to work, even among those who were previously unemployed for extended periods of time [52]. Cognitive and psychosocial barriers may be influential in whether patients were able to resume work. For instance, uncertainty or lack of intention to resume employment was the most significant predictor of delayed return to work, based on data from ACS and coronary artery bypass graft (CABG) patients [52]. A Cochrane Systematic Review examined interventions to promote return to work among patients with CAD based on 34 studies published between 1974–2017 [53]. The aggregate population also included those with obstructive CAD who had received surgical or interventional revascularization or had angiographic evidence of disease. There were no studies that assessed changes or interventions within the workplace. The authors concluded that cardiac rehabilitation programs, including exercise and counselling components, shortened return to work within the first 6 months after a cardiac event, but had little effect beyond this time [53]. More research with the (M)INOCA population is needed to accurately draw conclusions. One of the few in this area, a prospective study following 1-year outcomes at the multidisciplinary WHC in BC, Canada found that women with (M)INOCA who accessed the WHC had significant improvements in angina control, quality of life, depressive symptoms, and health care service burden [8]. A 3-year follow-up with this population found that women attending the WHC had significantly fewer emergency department visits for chest pain, from 92% of MINOCA patients having at least 1 visit in the preceding 3 years compared to 51% in the 3 years following enrollment [10]. The numbers decreased from 50% to 32% for INOCA patients, respectively [10].

The role of cardiac rehabilitation programs in facilitating return to work among women with (M)INOCA has yet to be elucidated, but they will likely require a multifactorial approach. In addition, program evaluation of workplacebased interventions and policies are not well-described in the literature. In a qualitative study of women with cardiac illness, common themes arising from their vocational impairment included feelings of guilt over unmet activity expectations, fatigue, depression, expressed desire to return to work and resume its social aspects, continued engagement in housework or unpaid work, and the struggle with role adjustment [17]. These findings suggest that comprehensive interventions need to address factors beyond lifestyle modification and be attuned to the meaning women with (M)INOCA ascribe to their illness experience.

### Measuring productivity loss in working women with (M)INOCA

To the authors' knowledge, there are no instruments that have been validated in patients with (M)INOCA to assess productivity or workplace impairment. The closest tool from the literature was a composite questionnaire designed in 1999 by Atienza et al. [54] to assess the quality of life in INOCA patients. The authors selected 33 questions from existing questionnaires and designed 29 new questions based on clinician interviews and patient observation. Scores were higher in patients with more frequent or severe chest pain episodes, indicating an inverse relationship with quality of life. The authors concluded their questionnaire had clinical validity, reliability, and responsiveness, as demonstrated by higher scores (meaning worse quality of life) in patients with symptomatic impairment [54].

There are existing tools with the potential to measure productivity outcomes in women with (M)INOCA, although they have not been formally evaluated in this population. The challenge with measuring productivity losses in women with (M)INOCA is that the extent of the problem is poorly defined. There is insufficient data to state that women with this diagnosis indeed suffer from impairment in the workplace, and the degree of meaningful variability in functional impairment due to other chronic diseases. Therefore, preliminary data must be collected to describe the employment trajectory of working women with (M)INOCA before a particular construct is identified as the focus. In addition, the natural history of the disease does not lend itself well to tools that measure absence or partial performance over a limited time course, such as days to weeks.

A particular questionnaire has promise in capturing the breadth of impairment experienced by this population of interest. Developed in BC, Canada, the *Valuation of Lost Productivity* (VOLP) is a validated composite questionnaire intended to measure the impact of health problems on productivity loss in time and monetary units [55,56]. It consists of 6 sections, including employment status, job characteristics, absenteeism, work performance, unpaid work, and working environment [55]. The VOLP has been used in populations with multiple sclerosis, CVD risk factors (but not formally diagnosed heart disease), rheumatoid arthritis, and caregivers for the ill or aged [56–59]. The VOLP focuses on valuation rather than measurement of time loss, and it is the only workplace questionnaire that assesses the economic repercussions of time lost from paid work at the societal level [55,56,59]. In addition, because the VOLP accounts for units of time lost even in non-paid work, such as childrearing or care for aging parents, it has the theoretical utility in providing information on the societal costs of (M)INOCA in the context of roles traditionally held by women of working age. Other strengths include its 3-month recall period for absenteeism, compared to the 1-week period in the *Work Productivity and Activity Impairment* (WPAI) questionnaire, another tool delivered alongside the VOLP to compare outcomes [55,59]. The 3-month time frame may be better suited to diseases that follow a similar chronological trajectory to (M)INOCA, where affected individuals are more likely to have been on long-term medical leave at baseline questionnaire administration. However, this extended time span introduces more recall bias. The questionnaire burden is an important consideration as well, particularly in patients who suffer comorbid fatigue, debilitating cognitive symptoms, or whose symptoms are worsened by psychosocial stress. Significant limitations are that neither the VOLP nor the WPAI have been formally validated in women with (M)INOCA. More research is needed before conclusions can be drawn about the validity of the constructs in each questionnaire as it relates to this population. Stakeholder input is crucial from both the patient and instrument developer perspective to ensure accurate interpretation of results.

## CONCLUSIONS

Nonobstructive coronary syndromes are associated with lower mortality rates and distinct pathophysiological profiles compared to obstructive syndromes. However, patients with a diagnosis that is categorized broadly as (M)INOCA experience substantial clinical, functional, and economic impairment. Women are disproportionately affected by (M)INOCA compared to men, highlighting the importance of sex-based differences in cardiac research. Trans and non-binary patients are significantly underrepresented in the literature. Despite growing recognition of (M)INOCA from a biomedical perspective, its economic and societal impacts are not well defined. Women with (M)INOCA may experience a high symptom burden, including chronic angina and functional impairment, leading to high health service utilization, repeat hospitalizations, and invasive diagnostic testing. This demographic faces significant productivity losses, with many leaving the workforce prematurely or experiencing prolonged periods of absenteeism or presenteeism.

There is a need for standardized and validated questionnaires on this topic to understand the directionality between work-related stressors and associated cardiac symptoms. This has implications for dedicated WHCs, through the potential to modify their programs to include return-to-work planning and career counselling. Current tools for assessing workplace impairment and productivity loss in this population are incomplete. The VOLP shows promise based on its use in populations with a female predominance and chronic disease trajectory, such as rheumatoid arthritis. Women enrolled in multidisciplinary WHCs have reported improvements in mental health and quality of life, but more studies are needed to evaluate the impact on work-related outcomes.

Single-center data supports the role of WHCs in reducing rates of acute care and emergency department visits, along with improved scores on scales for symptoms of depression and anxiety. However, it is still uncertain whether these outcomes are necessarily correlated with continued workforce participation. It would be useful to longitudinally track participation at WHCs alongside self-reported workplace impairment, corroborated by an objective measure such as number of hours worked weekly or number of missed workdays per month. The economic feasibility of consolidating multiple services related to women's heart health can be evaluated through quality improvement (QI) projects. Whether the health care savings of decreased emergency department visits outweigh the costs of center operation can be used as a template for establishing WHCs outside of Canada. Finally, the burden of (M)INOCA may be decreased by early identification and intervention in women with a strong risk factor profile or family history. It is challenging to extrapolate data on clinical or psychosocial outcomes in order to make broad conclusions around societal participation in the workforce, as it requires meticulous documentation of previous or current employment, and is subject to recall bias or discomfort with sharing confidential information. This approach requires input and collaboration between stakeholders at multiple levels, including employers, clinicians, policymakers, and patients.
